# Understanding the Relationship between Glutathione, TGF-β, and Vitamin D in Combating *Mycobacterium tuberculosis* Infections

**DOI:** 10.3390/jcm9092757

**Published:** 2020-08-26

**Authors:** Mohkam Singh, Charles Vaughn, Kayvan Sasaninia, Christopher Yeh, Devanshi Mehta, Ibrahim Khieran, Vishwanath Venketaraman

**Affiliations:** 1 Graduate College of Biomedical Sciences, Western University of Health Sciences, Pomona, CA 91766-1854, USA; mohkam.singh@westernu.edu (M.S.); charles.vaughn@westernu.edu (C.V.); kayvan.sasaninia@westernu.edu (K.S.); 2 College of Osteopathic Medicine of the Pacific, Western University of Health Sciences, Pomona, CA 91766-1854, USA; christopher.yeh@westernu.edu (C.Y.); devanshi.mehta@westernu.edu (D.M.); ibrahim.abukhieran@westernu.edu (I.K.)

**Keywords:** *Mycobacterium tuberculosis*, HIV, tuberculosis, TGF-β, vitamin D, glutathione

## Abstract

Tuberculosis (TB) remains a pervasive global health threat. A significant proportion of the world’s population that is affected by latent tuberculosis infection (LTBI) is at risk for reactivation and subsequent transmission to close contacts. Despite sustained efforts in eradication, the rise of multidrug-resistant strains of *Mycobacterium* *tuberculosis* (*M. tb*) has rendered traditional antibiotic therapy less effective at mitigating the morbidity and mortality of the disease. Management of TB is further complicated by medications with various off-target effects and poor compliance. Immunocompromised patients are the most at-risk in reactivation of a LTBI, due to impairment in effector immune responses. Our laboratory has previously reported that individuals suffering from Type 2 Diabetes Mellitus (T2DM) and HIV exhibited compromised levels of the antioxidant glutathione (GSH). Restoring the levels of GSH resulted in improved control of *M. tb* infection. The goal of this review is to provide insights on the diverse roles of TGF- β and vitamin D in altering the levels of GSH, granuloma formation, and clearance of *M. tb* infection. We propose that these pathways represent a potential avenue for future investigation and development of new TB treatment modalities.

## 1. *Mycobacterium tuberculosis* and the Host Immune Responses

### 1.1. Mycobacterium tuberculosis Infection

*Mycobacterium tuberculosis (M. tb)*, the causative agent for tuberculosis (TB), is an intracellular bacterial pathogen that typically colonizes the lower respiratory tract in humans. TB accounts for one of the top 10 causes of mortality worldwide from a single infectious agent. A major effort has been made in the 21st century to control and ultimately eradicate TB [1]. The efficacy of antimycobacterial medications are limited due to generally poor completion rates and undesired off-target effects like hepatotoxicity and neurological manifestations [2]. Additionally, the rise of multidrug-resistant strains of *M. tb* prompts the need for additional research to find alternative therapies to control this disease. The World Health Organization (WHO) reports that 1.7 billion people worldwide currently have latent *M. tb* infection (LTBI). Individuals with an LTBI are major reservoirs for the bacterium. Reactivation of the infection poses a threat to hosts, as well as to their contacts. Moreover, immunocompetent individuals with LTBI have a 5–10% lifetime risk for reactivation to active TB. Granuloma liquefaction is a common pathway for the reactivation of TB in immunodeficient or immunocompromised people [3].

Granulomatous lesions of the pulmonary parenchyma are clinical hallmarks of mycobacterial infection. A prospective cohort study found that patients with a history of active TB with these lesions were 5.4 times more likely to have pathological results on pulmonary function tests than those with LTBI [4].

The affected granulomas are leukocyte aggregates characterized by a monocytic cellular infiltrate. They commonly exhibit an epithelioid or multinucleated giant cell phenotype surrounded by CD4+ lymphocytes [5]. The transition to an interdigitated epithelioid phenotype creates a physical barrier which sequesters the pathogen at the site of infection, allowing selective monocyte infiltration of the lesion under the regulation of CD4+ lymphocytes [6]. This results in the inhibition of bacterial growth and localization of the inflammatory response to the site of infection by providing a microenvironment for continuous T-cell activation of infected macrophages [6,7].

However, there is mounting evidence that *M. tb* possesses metabolic adaptations specifically for maintaining virulence while in its dormant state. Upon contact with alveolar macrophages, cell surface receptor mediated recognition induces phagocytic uptake of *M. tb* and expresses proinflammatory cytokines [8]. At this stage, the macrophage does not undergo phagosome–lysosome fusion due to the presence of modified phospholipids in the *M. tb* cell wall such as lipoarabinomannan (LAM) and phosphatidylinositol mannoside (PIM). These bacterial compounds modulate cellular membrane trafficking mechanisms such that the pathogen continues to receive nutrients while simultaneously resisting lysosomal degradation [9].

### 1.2. Cytokines Profile in the Immune Response to M. tb

The persistence of intracellular *M. tb* in alveolar macrophages triggers the formation of the Th1 cells that produce cytokines including IL-2 and IFN-γ [10]. IFN-γ controls *M. tb* growth by promoting cell adhesion, apoptosis, cellular proliferation, and autophagy [11,12]. Another cytokine, TNF-α, secreted by macrophages, dendritic cells (DC), and T-cells, boosts the effector immune responses against *M. Tb* infection [13,14].

The anti-inflammatory cytokine TGF-β is well-documented as a major inhibitor of T-cell effector functions and the main mediator of fibrogenesis in the body [15,16]. Thus, *M. tb* infection in healthy subjects will result in a robust immune response via the release of these cytokines and formation of granulomas. However, individuals suffering from immunocompromising diseases, such as HIV or Type 2 Diabetes Mellitus (T2DM), will be at a higher risk for developing an active infection due to dysregulation of cytokine levels that may cause an impaired immune response [17]. Additionally, depletion of CD4+ cells during the advanced stages of HIV infection can lead to reactivation of a LTBI and increased susceptibility to reinfection [18].

### 1.3. The Role of Glutathione, TGF-B, and Vitamin D in the Immune Response

Glutathione (GSH) is a tripeptide of glutamine, cysteine, and glycine residues whose synthesis is catalyzed by the enzyme glutamyl-cysteine ligase (GCL). GSH is one of the most abundant intracellular thiols in metabolically aerobic cells and serves an important role in maintaining cellular redox homeostasis [19]. It is well documented as the most highly concentrated antioxidant within the cell [20]. Our lab has previously reported that H37Rv, a virulent laboratory strain of *M. tb,* can be completely cleared through supplementation with GSH or its precursor N-acetyl cysteine (NAC) in conjunction with first-line antibiotics; isoniazid, rifampin, or ethambutol; and within in vitro derived granulomas [21]. However, granulomas in the aforementioned study isolated from immunocompromised patients (uncontrolled T2DM) were not as robust as those from healthy subjects and were not able to limit growth of *M. tb*. This resulted in the intracellular survival of *M. tb* to be much higher in the immune cells derived from people with T2DM.

GSH levels have been shown to be significantly diminished in individuals with HIV infection and T2DM [22]. T2DM patients are observed to have increased levels of oxidative stress and decreased levels of GSH, accompanied by a downregulation in the expression of enzymes that are responsible for the de novo synthesis of GSH when compared to non-diabetic controls [23]. It has also been shown that, in these individuals, GSH decrease is likely due to increased levels of TGF-β which has been previously demonstrated to inhibit the rate-limiting enzyme, catalytic subunit of GCL (GCLC) that is responsible for GSH synthesis [24].

Another mechanism proposed to regulate GSH synthesis involves vitamin D. Upregulation of GCL in macrophages by vitamin D has been postulated to result in inhibition of oxidative stress and TGF-β in human bronchial epithelial cells in vitro [25]. Additionally, human monocytes supplemented with 1,25-(OH)_2_ vitamin D in vitro exhibited increased expression of the GCLC and increased formation of GSH [25]. The referenced studies demonstrate a cross-regulation of vitamin D, GSH synthesis, and activity of TGF-β. Prior to the widespread use of antibiotics for the treatment of TB, vitamin D was widely used in the treatment of active TB [26]. The immune-enhancing effects of vitamin D against *M. tb* infection, its interaction with TGF-β, and its modulatory effects on the levels of GSH are of great interest for investigation as an adjunct to current therapy.

### 1.4. Clinical Significance

In summary, individuals with weakened immune systems, such as those with T2DM and HIV, are increasingly susceptible to *M. tb* infection. T2DM is a systemic metabolic disease impacting a diverse array of tissues across multiple organ systems and is associated with comorbidities including, but not limited to, vascular disease and increased susceptibility to *M. tb* infection. It is estimated that 30 million people in the United States alone are affected by T2DM, with an additional 80 million in the early stage of pre-diabetes. It was reported that, in 2017, 20% of people with TB in the United States also had diabetes [27]. HIV impacts the immune response by infecting CD4+ T cells, thus augmenting an increase in the prevalence of acquired *M. tb* infection. The prevalence of both HIV and TB have been increasing worldwide and raise concern to those who are co-infected. The WHO states that the risk of developing TB is estimated to be between 16 and 27 times greater in people living with HIV than among those without HIV infection, and a study conducted in 2015 estimated 10.4 million cases of TB globally, including 1.2 million (11%) among people living with HIV [27]. The combination of TB and HIV infection raises major concerns for resource limited countries [28]. Therefore, an understanding of the pathways and commonalities that are responsible for the pathogenesis in TB, T2DM, and HIV and finding strategies to enhance immune responses in immunocompromised patients are urgently needed.

## 2. The Effects of Glutathione on the Immune System

### 2.1. The Role of GSH in Maintaining Cellular Redox Homeostasis

Reactive oxygen species (ROS) classically include the superoxide radical, hydroxyl radical, and hydrogen peroxide. These are highly reactive partially reduced intermediates generated by incomplete reduction of molecular oxygen in endogenous metabolic processes of aerobic organisms. ROS are partially responsible for clinical manifestations of fibrotic pulmonary diseases, an effect likely worsened by the close contact between respiratory tissue and the environment [29]. GSH functions to provide reducing equivalents for the reaction catalyzed by GSH peroxidase, which converts hydrogen peroxide to water and oxygen [19]. This reaction catalyzed by GSH peroxidase results in the conversion of GSH to its oxidized form glutathione disulfide (GSSG), which no longer possesses antioxidant capacity. The enzyme GSH reductase regenerates reduced GSH in a NADPH-dependent mechanism regulated via negative feedback by reduced GSH [30,31]. Maintenance of GSH concentration has a protective function against oxidative damage by ROS.

While some cells can uptake GSH from the extracellular space, most cells rely on de novo synthesis to maintain intracellular levels. GSH synthesis occurs in a two-step process catalyzed by GCL and GSH synthetase [32]. Activity of the GCL catalytic subunit (GCLC) is a major determinant of intracellular GSH concentration. It has also been shown that the concentration of GSH in the pleural fluid of patients with various fibrotic diseases, such as cystic fibrosis [33,34], idiopathic pulmonary fibrosis (IPF) [35,36,37,38,39,40,41], and sarcoidosis [42], is substantially decreased. Increased concentration of GSH has been demonstrated to promote Th1 cell differentiation via IL-12 and/or IL-27 [43]. While these cytokine responses are blunted in immunocompromised patients, TGF-β and other pro-inflammatory cytokines have been found to be overexpressed in individuals with T2DM [17].

### 2.2. Immunomodulatory Effects of GSH

In addition to its protective function against the oxidative stress, GSH has antimycobacterial and immunomodulatory activity. Whereas decreases in GSH appear to exacerbate some diseases like T2DM, TB, HIV, pulmonary fibrosis, and liver diseases, an increase in GSH levels has protective functions in these conditions [44]. Thus, levels of GSH can serve as an indicator of diseases via its role in the maintenance of redox balance [44]. Research has shown that GSH depletion is associated with increased free radicals, the production of pro-inflammatory cytokines, and the enhanced survival of intracellular *M. tb* [17,44,45,46,47,48,49]. One mechanism by which GSH serves its protective function is sequestration of nitric oxide (NO). GSH aids in the stabilization and delivery of a potent bactericidal free radical NO as S-nitrosoglutathione (GSNO) to *M. tb* internalized by macrophages and neutrophils in vitro [50].

Another important function of GSH is to support the cytolytic activity of natural killer (NK) cells. A study performed on NK cells demonstrated that administration of the GSH precursor N-acetyl cysteine (NAC) can lead to rescue of cytolytic activity after GSH depletion, thereby enhancing the function of NK cells against *M. tb* [51,52].

GSH is also shown to modulate the release of various cytokines. A study showed that treatment of whole blood with NAC causes an increase in IFN-γ, resulting in the enhancement of Th1 cell response against *M. tb* [12,53,54]. Studies have shown that higher Th1 and lower Th2 response helps control *M. tb* growth, supporting the importance of GSH levels in *M. tb* clearance and in modulating Th1 cytokines [54].

GSH is shown to play an important role in the maturation of antigen-presenting dendritic cells (DCs). Depletion of GSH in murine models also showed a lack of DC maturation, leading to decreased T-cell activation, Th1 effector response, and IL-12 production by DCs [55,56]. Another study performed on human DCs demonstrated that administration of the GSH precursor NAC inhibits activity of NF-κβ, a major immunomodulatory transcription factor.

## 3. TGF-β in the Immune Responses

### 3.1. Activation of TGF-β

Transforming growth factor β (TGF-β) is a peptide which performs an array of regulatory functions in cell proliferation, differentiation, and immunosuppression [29,57]. TGF-β also promotes the production of ROS and depletion of GSH [58,59]. The canonical pathway of TGF-β signaling is mediated by transmembrane heterodimers, each consisting of type I and type II TGF-β receptor monomers. Binding of TGF-β dimerizes two pairs of these heterodimers. The resultant tetrad propagates the signal cascade via a serine/threonine kinase pathway [60]. Smads comprise a family of structurally similar proteins that are the major intracellular signal transducers for TGF-β [61]. Activated Smad complexes are translocated into the nucleus where they may induce expression or repression of downstream elements, e.g., activating transcription factor (ATF) and NADPH oxidase (NOX) [62]. TGF-β has also demonstrated activity in non-Smad signaling pathways, including those of P13K/AktRK 1,2, p38, JNK, and NF-kB. These pathways have been investigated for their role in development of various neoplastic disorders [62]. The highly diverse spectrum of proliferative activity regulated by TGF-β poses a challenge to targeted pharmacological therapy due to its wide range of potential off-target effects.

### 3.2. TGF-β and GSH

Incubation with TGF-β is demonstrated to decrease GSH antioxidant activity via transcriptional downregulation of GCLC in murine hepatocytes. This results in TGF-β-induced apoptosis, which, in the same experiment, was shown to be abolished by overexpression of Bcl-XL [58]. TGF-β mediated downregulation of GCLC was abolished by inhibition of histone deacetylase, suggesting an epigenetic component to this pathway [58]. The promoter of GCLC gene is termed antioxidant response element (ARE) and is canonically under the control of the NFE2-related factor (Nrf2) pathway. Affinity of Nrf2 to ARE is heavily age-dependent in murine models, with older individuals exhibiting markedly decreased binding of Nrf2 to the GCLC nuclear response element [63]. Nrf2 transcriptional activity is tonically inhibited by Kelch-like erythroid cell-derived protein 1 (Keap1)-mediated ubiquitination and subsequent proteasomal degradation. The presence of ROS oxidizes the thiol groups of the Keap1-NrF2 complex, resulting in conformational changes that induce the dissociation and translocation of Nrf2 to the nucleus [64]. Nrf2 forms a heterodimeric complex with Maf on the ARE to increase the expression of several antioxidant enzymes, including GCLC, in order to synthesize GSH [65]. TGF-β also mediates post-transcriptional downregulation of GCLC in an NrF2-independent manner by inducing the expression of miR-433 miRNA targeting the 3′UTR of GCLC mRNA [66].

### 3.3. TGF-β and Tuberculosis

Incubation of human alveolar macrophages with *M. tb* results in increased production of TGF-β in vitro [67]. A case-control study performed in patients with acid fast bacilli-confirmed pulmonary TB or controls with other lung diseases, assayed fluid obtained from bronchoscopy with alveolar lavage (BAL). Results revealed that patients with *M. tb* infection had statistically significant increased expression of both TGF-β and TGF-β RI/RII receptors. IL-10, IL-2, and IFN-γ were also increased, as compared to control patients with other lung diseases [68]. TGF-β is well-documented for its role in pathogenesis of fibrotic disease of various organ systems, including the liver, kidney, and heart [69,70,71,72]. TGF-β activation induces the synthesis of extracellular matrix components and the epithelia–mesenchymal transition associated with tissue fibrosis [72,73]. TGF-β acts on macrophages to promote the production of ROS through the expression of NADPH oxidase (NOX) [69]. TGF-β has been shown as a major inhibitor of cytotoxic T-cell effector function and the main controller of fibrogenesis. This mechanism likely includes NOX4 as a downstream effector of TGF-β-induced fibrosis in response to increased oxidative stress. [37,74,75]. Another study showed that TGF-β1 selectively induces expression of the NOX4 isoform in human fetal lung mesenchymal cells and in myofibroblastic mesenchymal cells from patients with IPF [76]. Formation and healing of mature *M. tb* granulomas involves the differentiation of fibroblasts [7]. In macaques treated for TB with metronidazole, previously caseating granulomas progressed to centrally fibrotic appearance as they healed, and a fibrotic-to-fibrocalcific appearance was seen in completely healed lesions [77]. TGF-β receptor dominant negative transgenic mice demonstrate resistance to TB, which is abolished by addition of mesenchymal stem cells recruited by *M. tb* infection [78]. A study performed in patients with multidrug-resistant TB demonstrated that TGF-β plays a role in the expansion of Th17 cells in response to mycobacterial infection. This effect was shown to be dependent on co-expression of TGF-β with IL-23, as incubation with TGF-β without IL-23 resulted in decreased Th17 expression [4]. A separate study in murine models showed that both intranasal and subcutaneous administration of synthetic cyclic dinucleotide increased immunity upon challenge with virulent *M. tb* [79]. Additionally, cellular expression of the *M. tb* biomarker urokinase-plasminogen activator receptor is abolished by Smad3-siRNA in human monocytes [80]. The degree to which inhibition of TGF-β could interfere with the healing time is not yet understood and warrants further investigation. Taken together, these data suggest utility in manipulation of the TGF-β signaling cascade as a useful adjuvant to current pharmacologic therapy and vaccinations.

## 4. Vitamin D, TB, and the Immune Responses 

### 4.1. Cellular and Immunological Response to Vitamin D

The genomic actions of 1,25-(OH)_2_D are modulated through the vitamin D receptor (VDR), which is a transcription factor belonging to the steroid hormone receptor family [81]. This receptor targets genes that contain vitamin D response elements (VDREs) in their promoters, to which heterodimers of VDR and retinoid X receptors (RXRs) can bind and transactivate expression of the target genes [82]. VDR is expressed in at least 30 different target tissues [83,84]. Both malignant and nonmalignant cell types typically can express VDR and respond to 1,25-(OH)_2_D [82]. A study performed in both malignant and non-malignant human cell lines evaluated genome-wide analysis of DNA sequences with VDR-binding activity, which revealed consensus vitamin D response elements (VDRE) within the gene promoters for the innate antibacterial proteins cathelicidin (CAMP) and β-defensin 2 (DEFB4) [85]. The study also noted that CAMP appeared to be transcriptionally induced by 1,25-(OH)_2_D in monocytes [85]. Other studies have confirmed that vitamin D is critical for the regulation of both cathelicidins and β-defensins via CAMP and DEFB4, respectively, in both normal and transformed epithelial and hematopoietic cells [86,87,88,89]. This regulation is biologically important for the response of the innate immune system and may provide a useful mechanism to augment the immune response [90,91]. Human monocytes exhibit increased activation of antimicrobial activity against *M. tb* in human macrophages via a vitamin D-dependent pathway [92]. Research focusing on the expression of important antimicrobial peptide genes and activity are creating exciting discoveries revealing novel pathways regulated through vitamin D that could be essential for immune function and host-directed therapies.

TGF-β is a potent profibrogenic cytokine whose upregulation in fibrotic diseases makes it a key target for targeted therapy [71]. Among its inhibitory effects include the ability to decrease macrophage activation and effector function [69]. Notably, VDR has been shown to inhibit formation of ROS/RNS, as well as the epithelial–mesenchymal transition associated with TGF-β in human bronchial epithelial cells in vitro [93]. It has been postulated that this occurs via upregulation of GCLC in macrophages. Human monocytes supplemented with 1,25-(OH)_2_ vitamin D in vitro showed increased expression of GCLC and increased formation of GSH [93]. These important data represent an association between vitamin D supplementation and increased endogenous GSH synthesis, subsequent decreased ROS/RNS and profibrogenic activity of TGF-β. In light of the COVID-19 pandemic caused by the pathogen SARS-CoV-2, vitamin D is under investigation as a contributing factor to increased prevalence of COVID-19 in elderly populations and populations of low-socioeconomic status, with preliminary data suggesting an association [36,94].

### 4.2. Vitamin D and TB

Immunomodulatory properties of vitamin D, as well as its association with diet and sun exposure, have implicated its utility in addressing global health problems. Vitamin D was regularly used in the pre-antibiotic period to treat mycobacterial diseases such as TB and leprosy [26]. Moreover, 1,25-dihydroxyvitamin D (1,25-(OH)_2_D) and its prophylactic ability has long been thought to aid the innate and adaptive immune defenses against intracellular pathogens, although the association with its effects on *M. tb* infection is complex and not well described [95,96]. However, it is well documented that the immunocompromised population and patients with TB exhibit remarkably decreased levels of serum vitamin D [97]. There is often a positive correlation with ongoing TB infection and vitamin D insufficiency within afflicted populations. The extra-skeletal effects of vitamin D, including its immunomodulatory properties, are of great interest for today’s research on adjunctive host-directed therapies.

Apart from its known ability to regulate calcium homeostasis, vitamin D plays an important role in modulating the innate and humoral immune responses. There are two different forms in which Vitamin D can be obtained: vitamin D3 (also known as cholecalciferol) and vitamin D2 (or ergocalciferol). Cholecalciferol is actively synthesized in the skin via the exposure to UVB radiation, while ergocalciferol is synthesized in plants, yeast, and fungi. Vitamin D can be obtained in the diet from plants and animals or synthesized in the skin. Both forms are hydroxylated in the liver by the cytochrome P450 enzyme (CYP27A1) to 25-hydroxyvitamin D (25-[OH]D) in a substrate-dependent reaction [74]. This form of vitamin D (25-[OH]D) circulates in the blood by being bound to a vitamin D-binding protein [98]. Conversion of 25-(OH) D to 1,25 dihydroxyvitamin D (1,25-[OH]_2_D) allows for complete activation of the hormone. This is accomplished by mitochondrial 1-α-hydroxylase enzyme (CYP27B1). The majority of this conversion occurs in the renal tubules of the kidney; however, synthesis may also occur in other cells that express CYP27B1 [74].

## 5. HIV and TB

The ongoing prevalence of HIV is concerning health experts and has raised the need for continued research on ways to treat and manage this disease. Individuals suffering from HIV have an increased susceptibility to an active TB infection or the reactivation of latent TB [28]. Those infected with concomitant *M. tb* and HIV infection may experience accelerated deterioration of immunological functions [28]. Micronutrient deficiency appears to contribute to increased susceptibility to infection in HIV-positive patients. A large multinational cohort study of patients receiving antiretroviral therapy for HIV reported that deficiencies of vitamin D and vitamin A are associated with increased risk for developing pulmonary TB, with *p* = 0.03 and *p* = 0.01 respectively [99]. A double-blind placebo-controlled clinical trial has demonstrated that GSH depletion in individuals with HIV infection is associated with significant impairment of cytokine production that can be restored by oral administration of liposomal GSH [53].

Depletion of CD4+ T cells in HIV infection is a major contributing factor to immunocompromise. The number of HIV patients that are coinfected with *M. tb* is growing in prevalence and is a main concern for detrimental effects on the immune system. TB accelerates the progression of HIV infection, with an increased viral load, fall in CD4+ T cell count, and increased mortality [100]. TB infection affects an HIV infected individual by causing cytokine and chemokine irregularities that are believed to increase T-cell activation, enhance HIV replication, and result in a dysfunctional immune response [100]. Although HIV patients are more susceptible to TB regardless of CD4 T cell levels, the risk increases as CD4 T-cell levels decrease; these individuals are more likely to present with disseminated disease [101].

### GSH Supplementation in Increasing Cytokine Production in HIV Patients

A double-blind placebo-controlled clinical trial has demonstrated that GSH depletion in individuals with HIV infection is associated with significant impairment of cytokine production that can be restored by oral administration of liposomal GSH [53]. Decreased concentrations of GSH were observed in macrophages, NK cells, and T cells isolated from the peripheral blood of HIV-positive patients, as compared to controls. TGF-β and IL-10 were markedly elevated in HIV-positive individuals. Supplementation with oral liposomal GSH resulted in increased levels IL-2, IL-12, and IFN-γ, as well as decreased detection of the oxidative marker malondialdehyde. These changes were not observed in subjects taking placebo with empty liposomes [53]. Another clinical trial demonstrated 13 weeks of supplementation of oral liposomal Glutathione substantially decreased *M. tb* survival in in vitro infection assays performed on peripheral blood mononuclear cells isolated from individuals with HIV after 72 h of infection compared to empty liposome placebos [45].

## 6. Type-2 Diabetes Mellitus and TB

Type 2 Diabetes Mellitus is a prevalent metabolic disease throughout the globe. In 2018, the Centers for Disease Control (CDC) reported that 32.4 million people in the United States had diabetes and the WHO projects that diabetes will be the seventh leading cause of death by 2030 [102]. Uncontrolled TD2M may lead to impairment of the immune system, and this can increase the risk of TB for people with diabetes. Several studies have studied the correlation between T2DM and pulmonary TB. It was found that, in 2012, 50% of the patients that tested positive for *M. tb* infection had concomitant diabetes or pre-diabetes. In light of more recent events, the CDC reported, in 2017, that 20% of people with TB in the United States also had diabetes, making it an urgent need to understand the co-evolution of pathogenesis in T2DM and TB, and then develop strategies for enhancing the immune system.

Patients with T2DM are unable to produce sufficient insulin or develop insulin resistance, thus increasing the blood glucose levels. This excess glucose is not oxidized and gets shunted to the polyol pathway, where aldose reductase (AR) reduces glucose to sorbitol in the presence of its cofactor, NADPH. Therefore, as we see an increase in glucose shunt through the polyol pathway in T2DM, there is a simultaneous decrease in the available NADPH, but an increase in the production of advanced glycation end products (AGEs). This increase in AGEs results in oxidative stress via the generation of ROS [103,104]. Another factor that contributes to oxidative stress is the reduction in antioxidant capacity of GSH. Decreases in availability of NADPH in addition to overutilization of polyol pathways significantly decreases GSH levels and increases ROS levels [105]. People with T2DM, when compared to healthy individuals, have also been shown to have lower levels of GCLC, which is the rate-limiting enzyme in the synthesis of GSH [17]. In addition to this, patients with T2DM showed a reduction in the amount of GCLC that was correlated with increased amounts of TGF-β [17].

Cytokines IFN-γ and TNF-α, as shown in the previous sections, are imperative in the control and prevention of the spread of *M. tb*, respectively [24]. Individuals with T2DM show a significant decrease in these two cytokines, resulting in impaired immune responses against *M. tb*, which further results in active TB [24]. It has been shown in previous studies that an increase in glucose in the systemic circulation causes increases in the proinflammatory cytokines IL-1 and IL-6 [3,106]. These proinflammatory cytokines at high levels can inhibit the function of macrophages, making patients with T2DM more susceptible to *M. tb* infection. Ultimately, all these factors and dysregulations in the immune system of people with T2DM can result in a reactivation of *M. tb* in those with LTBI or primary infection, leading to active TB.

## 7. Conclusions

**Figure 1:**
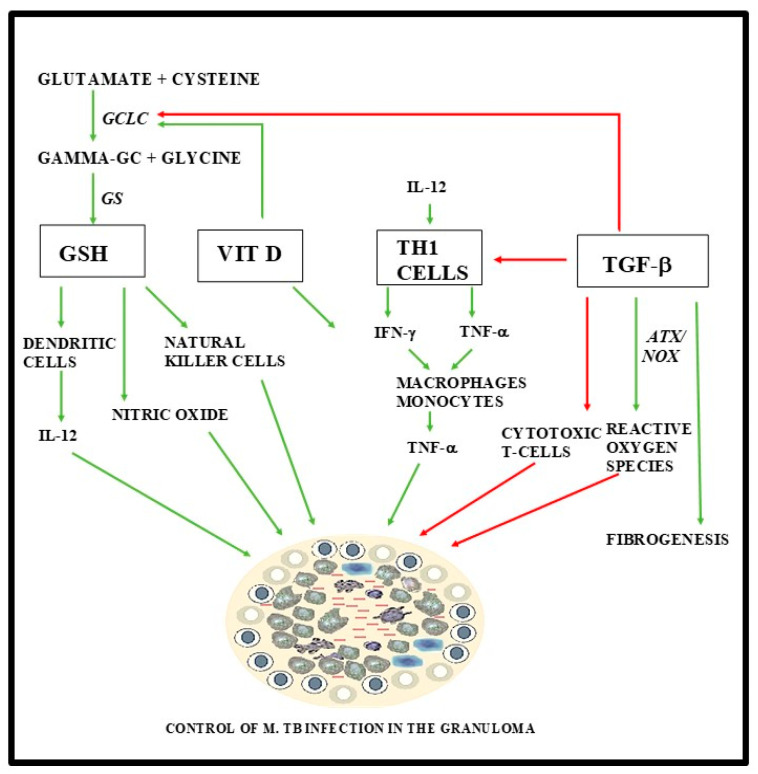
The diagram shows a summary of various factors that play a role in formation or inhibition of *Mycobacterium tuberculosis (M. tb)* granuloma. Green lines indicate stimulation, and red lines indicate inhibition.

The complex network of cellular signaling pathways associated with the mycobacterial granuloma represents a significant challenge in the management of pulmonary TB and LTBI. Comprehension of these mechanisms is imperative for the ultimate eradication of the disease, as proposed by the WHO. Here we present a potential avenue for development of new pharmacological interventions for TB. GSH plays a critical role in maintaining cellular redox homeostasis and in proliferating a host inflammatory response against pulmonary TB. In healthy individuals, *M. tb* is confined to a granuloma, resulting in a latent TB infection. Once a granuloma is formed, TGF-β initiates an immunosuppressive cascade to downregulate GSH through its catalytic subunit, GCLC, and initiate fibrogenesis (Figure 1) [73]. However, in immunocompromised patients, GSH levels are blunted and the proper immune response is not initiated. TGF-β downregulates the expression of GCLC, ultimately leading to decreased levels of GSH [58,107].

**Figure 2:**
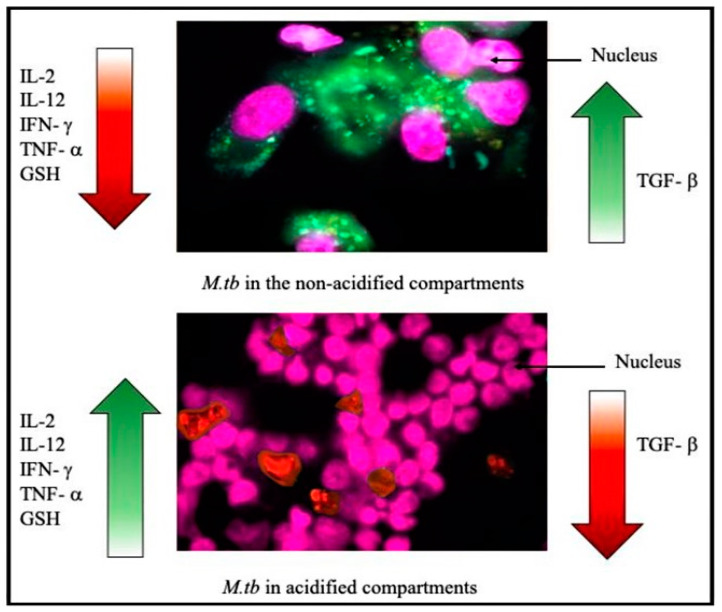
Quantification of *M. tb* in acidified and non-acidified compartments in granulomas from healthy subjects. *M. tb* expressing green fluorescent protein.

Our laboratory is exploring the effects of oxidative stress on an individual’s susceptibility to an active TB infection. We have observed that immunocompromised patients with HIV or T2DM have decreased levels of GSH, have increased levels of ROS, increased levels of proinflammatory and immunosuppressive cytokines such as TGF-β, and have an impaired ability to clear an *M. tb* infection in vitro. Supplementation with the GSH precursor NAC and standalone first-line antibiotics was sufficient in completely clearing *M. tb* infection within in vitro derived granulomas from T2DM individuals [21]. Immunocompromised patients have increased expression of TGF-β, which, in turn, counteracts the synthesis of GSH. This creates an environment more hospitable to an active *M. tb* infection (Figure 2).

Enhancing GSH through supplementation has been shown to inhibit intracellular *M. tb* growth [21]. Moreover, 1,25-(OH)_2_ vitamin D supplementation upregulates GCLC, enhancing GSH formation (Figure 1). The immunomodulatory effects of vitamin D supplementation may better enhance the effects of GSH supplementation, reducing the chance of an active TB infection in patients of immunocompromised status via manipulation of cytokine signaling pathways, notably that of TGF-β mediated fibrogenesis and immunosuppression. Taken together, we believe these findings carry potential as an avenue for further investigation in the development of new treatment modalities for pulmonary TB.
